# MiR-424-5p participates in esophageal squamous cell carcinoma invasion and metastasis via SMAD7 pathway mediated EMT

**DOI:** 10.1186/s13000-016-0536-9

**Published:** 2016-09-15

**Authors:** Feng Wang, Jun Wang, Xuan Yang, Danjie Chen, Liuxing Wang

**Affiliations:** 1 Department of Oncology, the First Affiliated Hospital of Zhengzhou University, Zhengzhou, 450052 People’s Republic of China; 2 Department of Microbiology and Immunology, College of Basic Medical Sciences, Zhengzhou University, Zhengzhou, 450052 People’s Republic of China

**Keywords:** Esophageal squamous cell carcinoma, miR-424-5p, SMAD7, Epithelial-mesenchymal transition

## Abstract

**Backgrounds:**

ESCC is a life-threatening disease due to invasion and metastasis in the early stage. Great efforts had been made to detect the molecular mechanisms which led to the invasion and metastasis in ESCC. Recent evidence had suggested that deregulation of miR-424-5p took an important role in cancers. However, its role and functional mechanism in ESCC had seldom been elucidated.

**Methods:**

The expression levels of miR-424-5p were detected in ESCC tissues and cell lines by real-time PCR methods. Then, the invasion, metastasis and proliferation ability of ESCC cell lines transfected with miR-424-5p mimics were analyzed separately by transwell invasion assay, wound healing assay and cell proliferation assay. Finally, the target gene of miR-424-5p was studied and verified by luciferase activity assay. And the role of miR-424-5p in EMT was also investigated by real-time PCR and western blot assay.

**Results:**

We showed that the expression levels of miR-424-5p were decreased both in ESCC tissues and cell lines. Furthermore, the expression levels of miR-424-5p were negatively linked to lymph node metastasis in ESCC tissues. Restoration of miR-424-5p in EC-1 cells by using miR-424-5p mimics could decrease the invasion, metastasis and proliferation of EC-1 cells, indicating its role in inhibition on the invasion and metastasis ability of ESCC cells and tissues. In addition, we demonstrated that SMAD7 was a specific target gene for miR-424-5p by luciferase activity assay and miR-424-5p could not only negatively regulate SMAD7 expression but also participate in EMT via SMAD7, because overexpression of SMAD7 could partly enhance the miR-424-5p anti-EMT function.

**Conclusions:**

Our results described that miR-424-5p -SMAD7 pathway contributed to ESCC invasion and metastasis and up-regulation of miR-424-5p perhaps provided a strategy for preventing tumor invasion, metastasis.

## Additional non-English language abstract

**目的:**食管癌因为早期侵袭转移的发生所以预后不佳。最近研究表明miR-424-5p表达下调在多种肿瘤的发生、发展过程中发挥重要作用。但其在食管癌发生、发展中的机制国内外未见报道。

**方法:**实时定量PCR法检测食管癌组织和细胞系中miR-424-5p的表达;合成miR-424-5p拟似物转染食管癌细胞系,侵袭小室、划痕试验和MTT试验检测miR-424-5p表达升高对食管癌细胞侵袭转移和增殖能力的影响;荧光素酶报告试验检测miR-424-5p下游靶基因;并进一步使用实时定量PCR和Western blot法检测miR-424-5p在食管癌上皮间质转化发生发展中的作用。

**结果:**miR-424-5p在食管癌组织和细胞系中的表达均明显下降,且其表达下降与食管癌组织的淋巴结转移密切相关。通过miR-424-5p拟似物增高miR-424-5p的表达可以降低食管癌细胞的体外侵袭转移和增殖能力。说明miR-424-5p在食管癌侵袭转移中发挥重要作用。荧光素梅报告试验证实SMAD7是miR-424-5p下游靶基因,miR-424-5p可以通过调节SMAD7的表达在食管癌的EMT发生发展中发挥作用。

**结论:**miR-424-5p -SMAD7途径在食管癌侵袭转移中发挥重要作用,升高miR-424-5p 的表达有可能成为食管癌治疗的手段之一。

## Background

Esophageal squamous cell carcinoma (ESCC) is one of the frequently occurring digestive malignant diseases in China [1, 2]. Patients with ESCC have higher mortality rates and 5-year survival rate is lower mainly because of local invasion, lymph node and distant metastasis [1]. Precise mechanisms of ESCC invasion and metastasis remain unclear, so further studies on the potential mechanisms involved in invasion and metastasis are critical for the improvement of prognosis for patients with ESCC.

MicroRNAs are about 22 nucleotides non-coding RNAs which can attach to their target mRNAs’ 3’UTR, by this way, to regulate the translation and stability of the target mRNAs through the action of the RNA-induced silencing complex [3–5]. In many kinds of cancers including ESCC, abnormal expression of microRNAs has been found. Furthermore, the abnormal expression of microRNAs has also been shown to be associated with tumor development [6, 7]. In addition, abnormal microRNAs expression has also been implicated in affecting metastatic and progression stage of cancers by the acquisition of metastatic potential [8–10].

MiR-424-5p is located on human chromosome Xq26.3, and recently has been classified in a large cluster together with miR-15/miR-16 [11]. However the expression of MiR-424-5p in different types of tumors suggested unequal roles. Zhang et al had demonstrated that miR-424-5p expression was significantly reduced in the liver cancer tissues compared with that of the corresponding non-cancerous liver tissues and down regulation of miR-424-5p in HCC tissues was also related to advanced disease progression in HCC patients [12]. While, Wu et al showed that miR-424-5p was significantly up regulated in pancreatic cancer [13].

Until now, great efforts had been made to identify the association between microRNAs expression and ESCC and to understand the functional role and molecular mechanism of aberrant-expressed microRNAs [14, 15]. The potential of some candidate microRNAs for clinical diagnosis and prognosis was revealed, and treatments involving microRNAs achieved some amazing curative effects in cancer disease models [16, 17]. However, the expression levels and role of miR-424-5p in ESCC had not been fully elucidated. Here, our findings provided evidence that miR-424-5p was a tumor suppressor gene in ESCC. Furthermore, we found that miR-424-5p perhaps played its role through negatively regulating SMAD7 signaling pathway. Taken together, miR-424-5p rescue might be a rational for diagnostic and therapeutic applications in ESCC.

## Methods

### Patients and tissue samples

Thirty-two pairs of ESCC tumor and adjacent normal mucosa in paraffin-embedded blocks were acquired from the First Affiliated Hospital of Zhengzhou University. No patients had received radiation therapy or chemotherapy before surgery. Two senior pathologists made and agreed upon the histological diagnosis of ESCC tissues. The paired ESCC tumor and adjacent normal mucosa areas of block were carefully dissected and transferred to RNase-free tube for RNA extraction.

### Cell culture and transfection

Esophageal squamous cell lines: EC9706, Eca109, EC-1 and immortal embryonic esophageal epithelium: SHEE cells were all cultivated in RPMI-1640 medium with 10 % FCS, at 37 °C in a 5 % CO_2_ humidified incubator. RPMI-1640 medium was replaced every 2–3 days. Transfection was typically carried out on cells that were 80 % confluent. Mature miR-424-5p mimics or control oligotides were purchased from Dharmacon Inc. 50 nM miR-424-5p mimics were transfected into EC-1 cells using Lipofectamine™ 2000 (Invitrogen) according to the manufacturer’s protocol. At the same time, EC-1 cells transfected with control oligotides or vacant EC-1 cells were used as negative or blank controls respectively. PcDNA3.1-SMAD7 transfection was done after the EC-1 cells trsfected with miR-424-5p mimics or control oligotides, EC-1 cells transfected with miR-424-5p mimics, pcDNA3.1-SMAD7 separately or vacant EC-1 cells were used as controls.

### RNA extraction

Total RNA, including microRNA was extracted from tumor and corresponding normal tissue in paraffin-embedded blocks using either TriReagent or mirVana miRNA isolation Kit according to the manufacturers protocol. The RNA was resuspended in 20 μl RNase-free water and stored at −20 °C. The quantity and quality of RNA were assessed by spectrophotometric and standard electrophoresis methods.

### Reverse transcription and real-time PCR

To examine the levels of miR-424-5p expression, TaqMan® miRNA Reverse Transcription kit were used to generate cDNA from miRNAs. Quantitative real-time PCR methods were used to measure the expression level of miR-424-5p using the TaqMan® MicroRNA Assay protocol specific for miR-424-5p. Relative expression values of miR-424-5p were calculated by the *CT*-based calibrated standard curve method. RNU6B miScript Primer Assay was used as an endogenous control. All of the experiments were done in triplicate.

To quantify the mRNA expression of SMAD7, vimentin, E-cadherin, transScript First-strand cDNA Synthesis SuperMix kit were used to generated cDNA from total RNA. Quantitative real-time PCR were then conducted using STBR Premix Ex Taq™ kit with the following primers: SMAD7 sense: GGAGTGGGGAGGAGTGAGTA, antisense: TCTTTTGTGGCCCACGTCTC; E-cadherin sense: ATGCTGATGCCCCCAATACC, antisense: GCTGTGAGGATGCCAGTTTC; vimentin sense: GGACCAGCTAACCAACGACA, antisense: AAGGTCAAGACGTGCCAGAG; β-actin sense: CTGAGGCTCTTTTCCAGCCT, antisense: CGCTCAGGAGGAGCAATGAT. The CT values were determined by setting a fixed threshold. The relative amount of mRNA was normalized to β-actin. All of the experiments were done in triplicate.

### Western blot assay

EC-1 cells in different groups were harvested and lysed for protein extraction. The concentration of protein was calculated with a BCA protein assay kit. Fifty microgram proteins were separated by SDS-PAGE, and the proteins were transferred by electro method to PVDF membranes. Subsequently, the PVDF membrane was blocked in 5 % fat-free milk for 2 h. Then, the PVDF membrane was incubated with the primary antibody against SAMD7, vimentin, E-cadherin and β-actin at 4 °C overnight. After that, the PVDF membrane were washed, and incubated with horseradish peroxidase-labelled secondary antibody for 1 h. After exposure and development, the protein expression was analyzed using a gel imaging analysis system. The Western blot assay was in triplicate.

### Transwell invasion assay

The invasion ability of EC-1 cells was measured by transwell invasion assay. Briefly, EC-1 cells in different groups cultivated with RPMI-1640 medium without FCS were put on the upper chamber coated with Matrigel. RPMI-1640 medium with 20 % FCS as chemoattractants were put to the lower chamber of the 24-well pates. After 48 h incubation, cotton swabs were used to wipe off the cells from the upper chamber. The number of cells migrating to the lower chamber was calculated by inverted microscopy after hematoxylin and eosin (HE) staining. And the number of and the mean of number of cells in each field represented the invasive ability of the cells. All of the experiments were done in triplicate.

### Wound healing assay

EC-1 cells were seeded into 12-well plates and cultured to 70 % confluence. One millimeter width wounds were made with a plastic tip, and EC-1 cells were cultivated in a serum-free RPMI-1640 medium. EC-1 cells migrated into wound place, then after 48 h the average distance of migrating cells was measured under an inverted microscopy.

### Cell proliferation assay

Cell proliferation ability was measured using the MTT assay. In brief, cells were placed into 96-well plates (BD Biosciences) at a cell density of 1 × 10^3^ cells/well in growth medium. 24, 48 and 72 h after transfection, 100 μl of fresh serum-free RPMI-1640 medium with 0.5 g/l MTT replaced RPMI-1640 medium in each well. After incubation for 4 h at 37 °C, the MTT medium was removed and 50 μl of DMSO was added to each well. The staining intensity in each group was measured by detecting the absorbance at 450 nm. All of the experiments were done in triplicate.

### Luciferase activity assay

The pmir-REPORT system was used to determine whether SMAD7 was a target of miR-424-5p. The wild-type SMAD7 3’UTR luciferase reporter vector (pmirGLO-SMAD7-WT) was constructed by amplifying 3’UTR of the SMAD7 gene and then cloning them into the Hind III and Spe I sites of pmirGLO-control vector and amplified in chemically competent DH5α. Site-directed mutagenesis kit (Promega) were used to made the mutant type of SMAD7 3’UTR luciferase reporter vector (pmirGLO-SMAD7-MUT). All constructs were verified by sequencing. MiR-424-5p mimics or control oligotides, 100 ng luciferase reporter plasmid were cotransfected into EC-1 cells in 6-well plates. After 24 h, cells were lysed and luciferase activity was measured using a Dual-Luciferase Reporter Assay System (Promega).

### Statistical analysis

The database was determined by the SPSS 11.0 software for analysis. Data were showed as mean ± SD. Student’s *T*-test was always performed between two sets of data. The criteria for *T*-test significance was based on the two-tailed distribution and for paired or equal variance type. A *p*-value of <0.05 was viewed as statistically significant.

## Results

### MiR-424-5p was down-regulated in ESCC tissues and ESCC cell lines

The biological roles of miR-424-5p in ESCC progression had not been fully delineated. To define the potential role miR-424-5p, we detected the expression levels of miR-424-5p in ESCC tissues and ESCC cell lines.

**Fig. 1:**
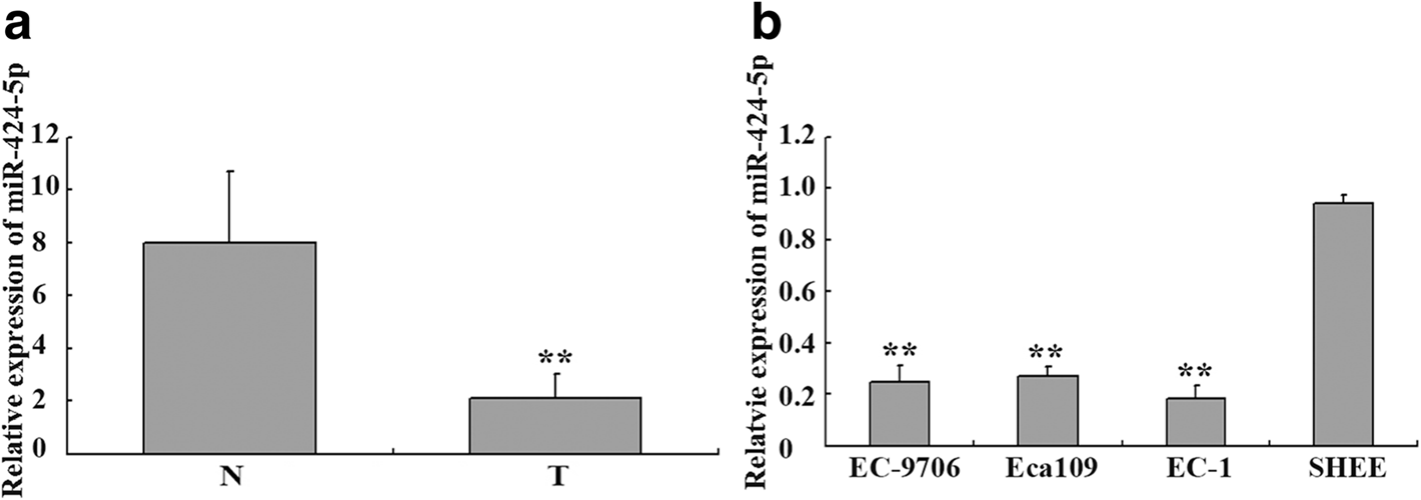
Downregulation of miR-424-5p in ESCC tissues and cell lines. **a** The expression levels of miR-424-5p were analyzed in ESCC tissues (T) and adjacent normal mucosa tissues (N) by real-time PCR method, RNU6B levels were used as an internal control. And the results of real-time PCR assay revealed down-regulation of miR-424-5p in ESCC tissue compare with adjacent normal mucosa tissues. **p < 0.01. **b** The expression levels of miR-424-5p were analyzed in ESCC cell lines (EC9706, Eca109 and EC-1) and immortal embryonic esophageal epithelium cell line (SHEE) by real-time PCR method, RNU6B levels were used as an internal control. Expression of miR-424-5p was down-regulated in EC9706, Eca109 and EC-1 compared with SHEE. **p < 0.01. The expression of miR-424-5p in EC-1 was the lowest in three kinds of ESCC cell lines although there had no statistical difference among them

First, we compared the expression of miR-424-5p in ESCC tissues (*n* = 32) and adjacent normal mucosa tissue (*n* = 32). And the expression of miR-424-5p was significantly decreased in ESCC tissues (Fig. 1a). To further explore the clinical value of miR-424-5p expression, the relation between the expression of miR-424-5p and clinicopathological parameters of patients with ESCC were investigated. As shown in Table 1, expression levels of miR-424-5p did not appear association with gender, age or invasion depth (*p* > 0.05). Nevertheless the expression of miR-424-5p was negatively linked to differentiation and lymph node metastasis (*p* > 0.05). So in ESCC tissues, we came to the conclusion that down-regulated miR-424-5p had a negative trend with the invasion and metastasis of ESCC.

**Table 1:** Relationship between miR-424-5p expression and clinico-pathological parameters

Clinico-pathological parameters	Number	Mean ± SD	*p* value
Age (years)			
≥ 60	25	9.36 ± 3.20	0.421
< 60	7	9.82 ± 2.91	
Gender			
Male	19	8.96 ± 2.79	0.518
Female	13	9.43 ± 3.01	
Depth of invasion			
Superficial	12	10.85 ± 1.77	0.018
Deep	20	7.32 ± 0.97	
Lymph node metastasis			
No	14	11.66 ± 2.95	0.006
Yes	18	7.11 ± 2.62	
Differentiation			
Well differentiated	17	10.09 ± 2.32	0.095
Moderately/poorly differentiated	15	9.73 ± 1.69	

The miR-424-5p expression was associated with depth of invasion and lymph node metastasis. *P* < 0.05

To further confirm the low-expression trend of miR-424-5p in the ESCC tissues, the expression of miR-424-5p was detected by quantitative real-time PCR in ESCC cell lines(EC9706, Eca-109, EC-1) and SHEE cells (immortal embryonic esophageal epithelium). Although miR-424-5p expression could be found in three ESCC cell lines, their expression were all significantly lower than that in SHEE cells (*p* < 0.05) (Fig. 1b). Thus, these results demonstrated that miR-424-5p was also significantly down-regulated in ESCC cell lines. We chose EC-1 cells which showed the lowest miR-424-5p expression for further experiments in this study.

### Overexperssion of miR-424-5p inhibited EC-1 cells invasion and metastasis

**Fig. 2:**
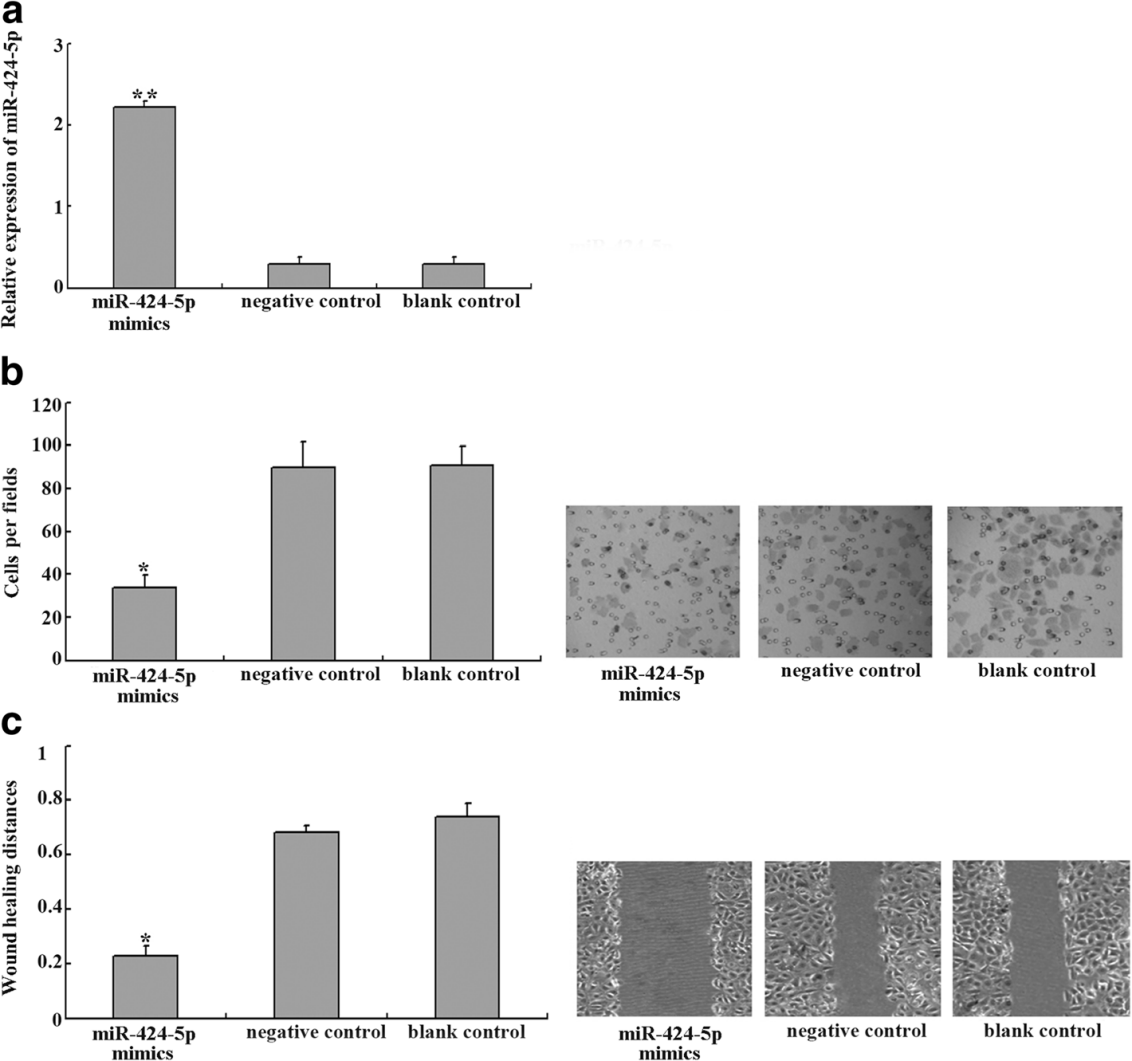
Overexperssion of miR-424-5p inhibited EC-1 cells invasion and metastasis. **a** Real-time PCR analysis of miR-424-5p in EC-1 cells transfected with miR-424-5p mimics showed increased miR-424-5p expression compared with EC-1 cells transfected with those in negative control or blank control. **p < 0.01. **b** The results of cell invasion assay showed that the average number of invasive cells in miR-424-5p mimics groups were greatly lower than those in negative control or blank control. *p < 0.05. Representative fields of invasive cells on the membrane were shown. **c** The results of wound healing assay also showed that the wound healing ability of EC-1 cells transfected with miR-424-5p were significantly increased compared with EC-1 cells transfected with those in negative control or blank control. *p < 0.05. Representative pictures of wound healing distance were shown

To validate the function of miR-424-5p, we transient transfected miR-424-5p mimics (mimic the endogenous mature miR-424-5p function) or control oligotides into EC-1 cell. Forty-eight hours after transfection, miR-424-5p expression in EC-1 cells showed a dramatically increase compared to that in negative control or blank control (*p* < 0.05) (Fig. 2a). No statistical significance of miR-424-5p expression was found between the negative and blank control groups (*p* > 0.05). These results indicated that miR-424-5p mimics could regulate miR-424-5p expression effectively in EC-1 cells.

As mentioned above, the expression levels of miR-424-5p were negatively correlated with differentiation and lymph node metastasis in ESCC tissues. To further study on the influence of miR-424-5p on EC-1 invasion and metastasis ability, then several functional analyses were performed. The results of transwell invasion assay showed that overexpression of miR-424-5p significantly decreased the invasiveness of EC-1 cells compared to those in control groups (*p* < 0.05) (Fig. 2b). Furthermore, the wound healing ability of EC-1 cells transfected with miR-424-5p mimics were also decreased greatly (*p* < 0.05) (Fig. 2c). These results showed that miR-1195 might be an important element inhibiting invasion and metastasis of EC-1 cells.

### Overexperssion of miR-424-5p inhibited ESCC cells growth

**Fig. 3:**
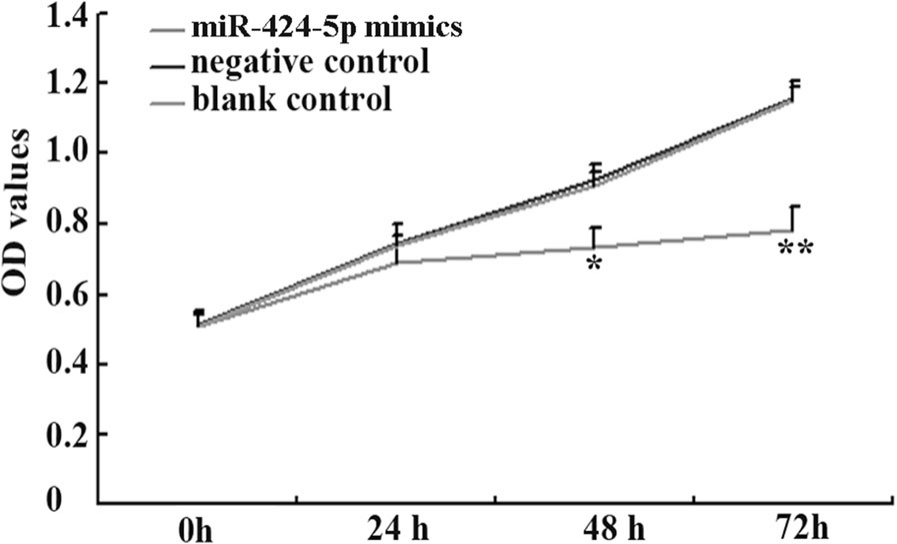
Overexperssion of miR-424-5p inhibited EC-1 cells proliferation ability. MTT viability assays were performed 24, 48 and 72 h after the transfection of miR-424-5p. And the results showed that the proliferation ability of EC-1 cells transfected with miR-424-5p were greatly lower than those in negative control or blank control. **p < 0.01, *p < 0.05

Inhibition of cell invasion and metastasis in cancer cells is usually associated with cancer cells abnormal growth. Here, we demonstrated the effects of miR-424-5p on the proliferation of EC-1 cells after transfected with miR-424-5p mimics. And the results of cell proliferation assay showed that in EC-1 cells, starting at 48 h, there was an decrease in cell proliferation with overexpression of miR-424-5p relative to negative or blank control (*p* < 0.05) (Fig. 3). These data suggested a specific role for miR-424-5p in driving EC-1 cells proliferation.

### MiR-424-5p targets SMAD7 leading to inhibition of the TGF-β-SMAD7 pathway

**Fig. 4:**
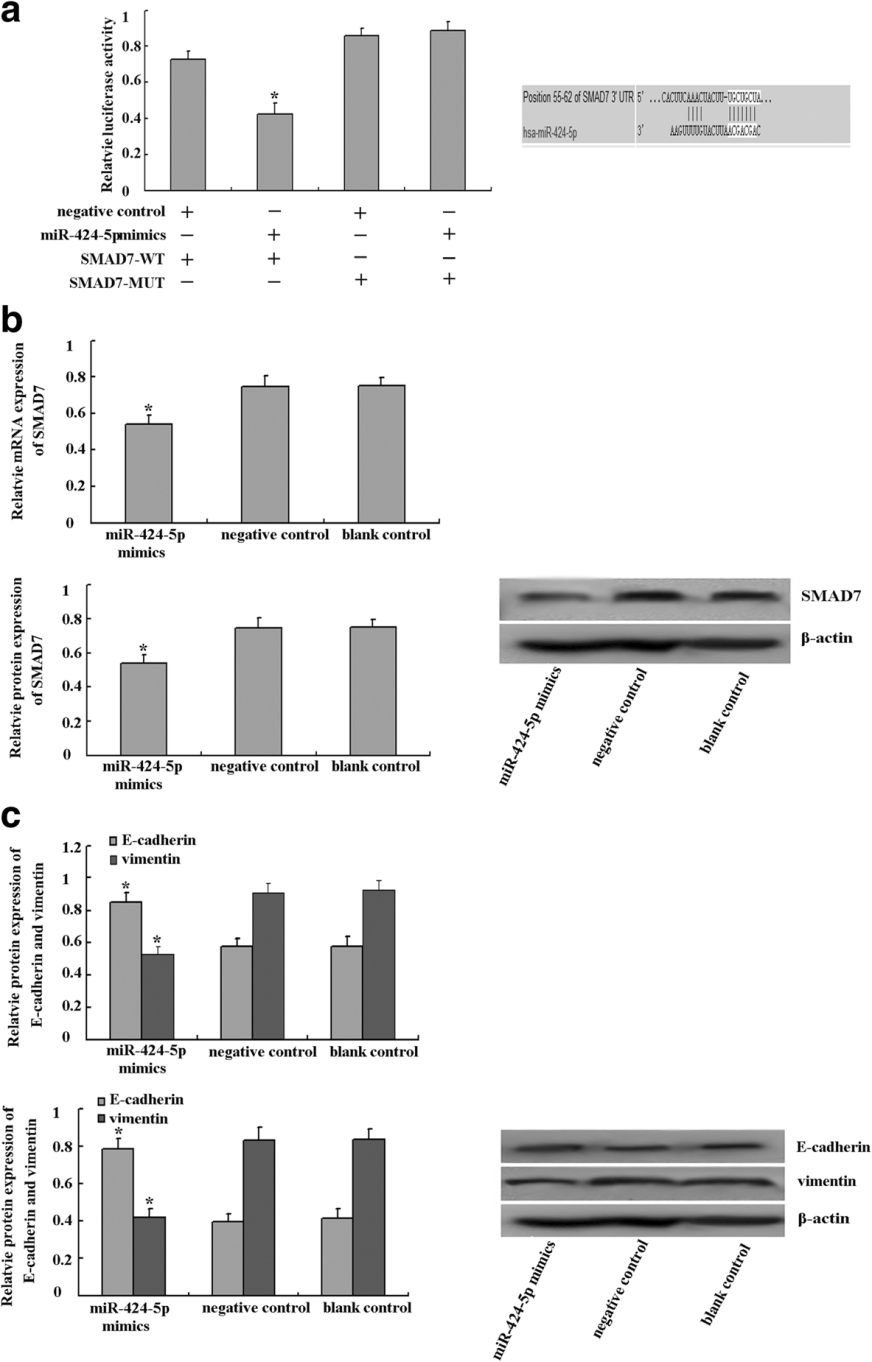
MiR-424-5p targeted SMAD7 by binding to its 3’UTR. **a** The SMAD7 3’UTR was a potential target of miR-424-5p. MiR-424-5p mimics decreased luciferase activities controlled by wild-type SMAD7-3’UTR *p < 0.05, while did not influence the luciferase activities controlled by mutant SMAD7-3’UTR or miR-424-5p negative control. **b** SMAD7 mRNA and protein expression in EC-1 cells transfected with miR-424-5p mimics were detected by real-time PCR and western blot assay 48 h after transfection, EC-1 cells transfected with control oligotides or vacant EC-1 cells were used as negative or blank controls respectively. β-actin was used as an internal control. And the results showed that the mRNA and protein expression of SMAD7 were significantly decreased compared to those in control groups. *p < 0.05. **c** Expression of miR-424-5p mimics also enhanced the expression of E-cadherin and suppressed the expression of vimentin. *p < 0.05

MiR-424-5p was frequently down–regulated in ESCC and was a key factor in cell invasion, metastasis and proliferation as demonstrated above. We then observed the molecular mechanism of miR-424-5p mediated biological function. We examined its potential targets by searching Targetscan database and PicTar and found that among the candidate targets, the 3’-UTR of SMAD7 contained the miR-424-5p conserved binding site (ACGACGA). Furthermore, in order to examine whether SMAD7 was a functional target of miR-424-5p, we evaluated the reporter activity in cells co-transfected with miR-424-5p mimics (or negative control) and pmirGLO-SMAD7-WT (or pmirGLO-SMAD7-MUT). As shown in Fig. 4a, EC-1 cells co-transfected with miR-424-5p mimics and pmirGLO-SMAD7-WT demonstrated a significant decrease of reporter activity in comparison with those co-transfected with negative control and pmirGLO-SMAD7-WT(*p* < 0.05). In addition, EC-1 cells co-transfected with miR-424-5p mimics and pmirGLO-SMAD7-MUT or negative control and pmirGLO-SMAD7-MUT showed no significant decrease of reporter activity in comparison with those co-transfected with negative control and pmirGLO-SMAD7-MUT(*p* > 0.05). Taken together, luciferase activity of wild-type SMAD7-3’-UTR but not mutated SMAD7-3’-UTR reporter was decreased by miR-424-5p mimic in EC-1 cells. Furthermore, the western blot assay and real-time PCR analyses revealed that SMAD7 protein and mRNA expression were greatly decreased in miR-424-5p mimics transfected EC-1 cells (Fig. 4b). All these results revealed that SMAD7 is a target of miR-424-5p in ESCC.

The SMAD7 signaling pathway is known to facilitate metastasis in advanced malignancy by taking part in EMT processes. In the present study, two major genes: E-cadherin and vimentin involved the EMT processes were also analyzed by western blot and real-time PCR. As shown in Fig. 4c, the mRNA and protein expression of E-cadherin were increased in EC-1 cells transfected with miR-424-5p mimics while the mRNA and protein expression of vimentin were decreased compared to those in negative or blank control (all *p* < 0.05). These data elucidated to us that miR-424-5p could participate in EMT in ESCC cells and perhaps this participation were mediated by the SMAD7 signaling pathway.

### Overexpression of SMAD7 could partially enhance the EMT weaken by higher expression of MiR-424-5p

**Fig. 5:**
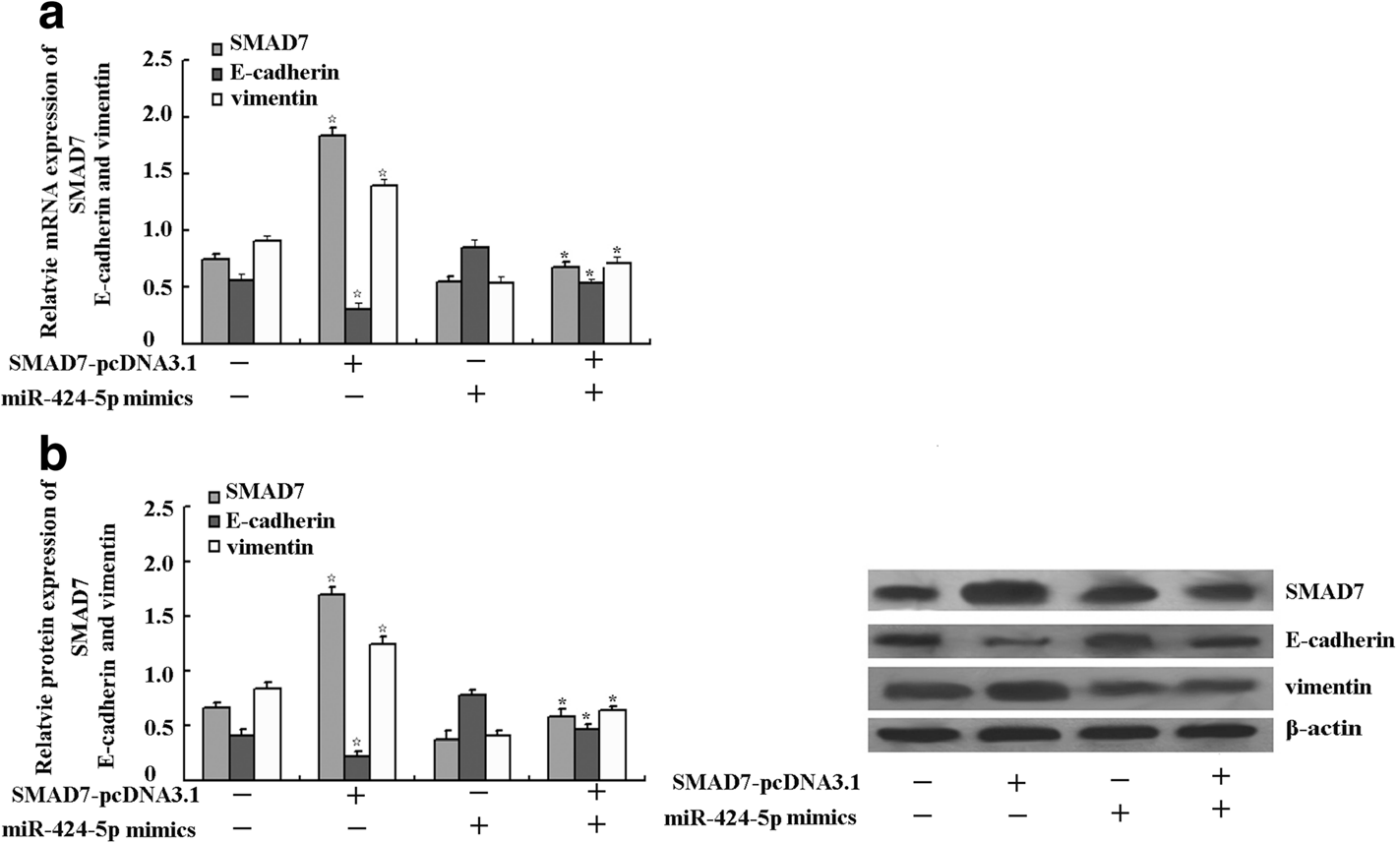
**a b** Real-time PCR and western blot analysis showed that the SMAD7 mRNA and protein expression in EC-1 cells transfected with pcDNA3.1-SMAD7 increased greatly compared to blank EC-1 cells. ☆ < 0.05 vs blank control. Furthermore, pcDNA3.1-SMAD7 transfection could partially enhance the EMT decreased by miR-424-5p mimics transfection by increasing the mRNA and protein expression of E-cadherin, while decreasing the mRNA and protein expression of vimentin. *p < 0.05 vs miR-424-5p mimics

In order to give further proof of the role of SMAD7 signaling pathway in miR-424-5p involved EMT, SMAD7 eukaryotic expression vector pcDNA3.1-SMAD7 was transfected into EC-1 cells for 48 h after the EC-1 cells were treated with miR-424-5p mimics or control oligotides. As shown in Fig. 5a, b, the overexpression of SMAD7 could partially reverse the expression of E-caherin and vimentin treated with miR-424-5p mimics. Thus, our results further proved that SMAD7 signaling pathway participated in the miR-424-5p regulated EMT in esophageal squamous cell carcinoma.

## Discussion

Lately, miRNAs have been shown to regulate tumor invasion, metastasis, providing for us a new view on the invasion process [18]. MiR-424-5p had been showed to participate in various cellular processes especially the invasion and metastasis processes [12]. Nonetheless, there had been no reports on the expression and role of miR-424-5p in ESCC.

In this study, the expression of miR-424-5p was examined in paraffin-embedded blocks. We showed that the expression of miR-424-5p were greatly lower in ESCC tissues compared with those in adjacent normal mucosa tissues. Furthermore, the three kinds of ESCC cell lines (EC9706, Eca109 and EC-1) we chose were also showed lower expression levels of miR-424-5p compared to that in SHEE cells, stating clearly that the loss of miR-424-5p might be a common event in tumorigenesis. Strikingly, the expression of miR-424-5p were also greatly lower in ESCC tissues with lymph node metastasis, compared to that without lymph node metastasis, indicating that the expression of miR-424-5p might be negatively linked to metastasis in ESCC.

Based on these, we supposed that miR-424-5p might play an important role as a tumor suppressor in the invasion and metastasis in ESCC. We then performed functional analyses to study on the role of miR-424-5p in the invasion and metastasis ability of EC-1 cells (expressed the lowest miR-424-5p levels in the three kinds of ESCC lines) transfected with miR-424-5p mimics. As expected, in our real-time PCR analysis of the EC-1 cells, miR-424-5p expression was markedly up-regulated in EC-1 cells transfected with miR-424-5p mimics compared to those in negative and blank control. In addition, miR-424-5p overexpression dramatically inhibited EC-1 cells invasion and metastasis ability. Cell proliferation ability of EC-1 cells transfected with miR-424-5p mimics was also decreased. These results further validated our suggestion that miR-424-5p might function as a tumor suppressor and played an important role in inhibition on the invasion and metastasis of ESCC cells. By detecting the expression of miR-424-5p can perhaps help us to tell the possibility of invasion and metastasis.

As part of our research on how the down-regulation of miR-424-5p influenced EC-1 cells invasion and metastasis, we used TargetScan and PicTar to identify target genes of miR-424-5p. This approach allowed us to verify SMAD7 as a potential target of miR-424-5p. We performed 3’UTR luciferase assay, and observed that luciferase activity was decreased after co-transfection of miR-424-5p mimics and a pmirGLO-SMAD7-WT. We also demonstrated that restoration of miR-424-5p expression levels tightly regulated SMAD7 expression and further confirmed that SMAD7 as a direct target of miR-424-5p. SMAD7 was initially isolated as an oncogene and was the downstream mediator of transforming growth factor beta (TGF-β), which is an important multifunctional cytokine that regulates cell proliferation and progression [19–21]. TGF-β-SMAD7 signaling pathway has been showed to be a key signaling pathway of epithelial-mesenchymal transition (EMT) in cancers [22, 23]. EMT was viewed as the first step in cancer invasion and metastasis, a number of evidences had recently confirmed that some microRNAs were involved in this process [24]. In the current study, we demonstrated that with the restoration of miR-424-5p, the expression of epithelial marker E-cadherin which was also a hall marker of the occurrence of EMT was increased while the expression of mesenchymal marker vimentin decreased. In addition, overexpression of SMAD7 could enhance the EMT weakened by miR-424-5p mimics. These results indicated that miR-424-5p could also take part in EMT in ESCC cells and miR-424-5p perhaps participated in EMT in ESCC cells via the SMAD7 signaling pathway.

## Conclusion

Our data indicated that down-regulated miR-424-5p was implicated in ESCC tissues and cell lines, particularly in ESCC tissues with lymph node metastasis. In addition, we demonstrated the function role of miR-424-5p in invasion and metastasis in EC-1 cells by up-regulating miR-424-5p expression levels in EC-1 cells. Finally, we elucidated overexpression of miR-424-5p decreased EC-1 cells invasion and metastasis through mechanisms involving SMAD7 signaling pathway and EMT. We are now investigating the role of SMAD7 signaling pathway in miR-424-5p mediated EMT and the potential role of miR-424-5p as prognostic and predicative biomarkers in ESCC.

## Abbreviations

DMSO: Dimethyl sulfoxide
EC-1: Esophageal cancer cell line 1
EC9706: Esophageal cancer cell line 9706
Eca109: Esophageal cancer cell line 109
EMT: Epithelial mesenchymal transition
ESCC: Esophageal squamous cell cancer
FCS: Fetal calf serum
HCC: Hepatocellular Carcinoma
HE: Hematoxylin and eosin
miR: MicroRNA
MTT: 3-(4,5-Dimethylthiazol-2-yl)-2,5-diphenyltetrazolium bromide
PVDF: Polyvinylidene fluoride
SHEE: Immortal embryonic esophageal epithelium
SMAD7: Sekelsky mothers against dpp 7
SPSS: Statistical package for social science
TGF-β: Transforming growth factor-β
UTR: Untransliated region
